# First detected geographical cluster of BoDV-1 encephalitis from same small village in two children: therapeutic considerations and epidemiological implications

**DOI:** 10.1007/s15010-023-01998-w

**Published:** 2023-02-23

**Authors:** Leonie Grosse, Victoria Lieftüchter, Yannik Vollmuth, Florian Hoffmann, Martin Olivieri, Karl Reiter, Moritz Tacke, Florian Heinen, Ingo Borggraefe, Andreas Osterman, Maria Forstner, Johannes Hübner, Ulrich von Both, Lena Birzele, Meino Rohlfs, Adrian Schomburg, Merle M. Böhmer, Viktoria Ruf, Dániel Cadar, Birgit Muntau, Kirsten Pörtner, Dennis Tappe

**Affiliations:** 1 Department of Pediatrics, Dr. Von Hauner Children’s Hospital, Ludwig-Maximilians-University, Lindwurmstr. 4, 80377 Munich, Germany; 2 Center for Children with Medical Complexity – iSPZ Hauner, Ludwig-Maximilians-University, Munich, Germany; 3 Max-Von-Pettenkofer Institute, Ludwig-Maximilians-University, Munich, Germany; 4 German Center for Infection Research (DZIF), Partner Site Munich, Munich, Germany; 5 Department of Physiological Chemistry, LMU Biomedical Center Munich, Ludwig-Maximilians-University, Munich, Germany; 6 Department of Infectious Disease Epidemiology, Bavarian Health and Food Safety Authority, Munich, Germany; 7 Institute of Social Medicine and Health Systems Research, Otto-Von-Guericke-University, Magdeburg, Germany; 8 Center for Neuropathology and Prion Research, Ludwig-Maximilians-University, Munich, Germany; 9 Bernhard Nocht Institute for Tropical Medicine, Bernhard-Nocht-Str. 74, 20359 Hamburg, Germany; 10 Department of Infectious Disease Epidemiology, Robert Koch Institute, Berlin, Germany

**Keywords:** Borna disease virus, Bornavirus, Treatment, Transmission, Epidemiology, Immunosuppression

## Abstract

**Background:**

The Borna disease virus (BoDV-1) is an emerging zoonotic virus causing severe and mostly fatal encephalitis in humans.

**Methods and Results:**

A local cluster of fatal BoDV-1 encephalitis cases was detected in the same village three years apart affecting two children. While the first case was diagnosed late in the course of disease, a very early diagnosis and treatment attempt facilitated by heightened awareness was achieved in the second case. Therapy started as early as day 12 of disease. Antiviral therapy encompassed favipiravir and ribavirin, and, after bioinformatic modelling, also remdesivir. As the disease is immunopathogenetically mediated, an intensified anti-inflammatory therapy was administered. Following initial impressive clinical improvement, the course was also fatal, although clearly prolonged. Viral RNA was detected by qPCR in tear fluid and saliva, constituting a possible transmission risk for health care professionals. Highest viral loads were found *post mortem* in the olfactory nerve and the limbic system, possibly reflecting the portal of entry for BoDV-1. Whole exome sequencing in both patients yielded no hint for underlying immunodeficiency. Full virus genomes belonging to the same cluster were obtained in both cases by next-generation sequencing. Sequences were not identical, indicating viral diversity in natural reservoirs. Specific transmission events or a common source of infection were not found by structured interviews. Patients lived 750m apart from each other and on the fringe of the settlement, a recently shown relevant risk factor.

**Conclusion:**

Our report highlights the urgent necessity of effective treatment strategies, heightened awareness and early diagnosis. Gaps of knowledge regarding risk factors, transmission events, and tailored prevention methods become apparent. Whether this case cluster reflects endemicity or a geographical hot spot needs further investigation.

## Introduction

The Borna disease virus 1 (BoDV-1) is a zoonotic virus of the *Bornaviridae* family that is harbored at least by the bicolored white-toothed shrew (*Crocidura leucodon*) as a natural reservoir [1–3]. In 2018, BoDV-1 was shown to cause severe and mostly fatal encephalitis in humans in Germany [4, 5]. The virus is non-cytopathogenic and the disease is assumed to be immunopathogenetically mediated [6], showing severe cytokine dysregulation in patients [7], accompanied by strong microglia and astrocyte activation [7, 8]. Currently, more than 40 human BoDV-1 encephalitis cases are registered in Germany, most of them were retrospectively diagnosed. The direct detection of zoonotic bornaviruses became notifiable in Germany in 2020. In 2021, the so far yearly maximum of seven acute cases were reported to public health authorities. Most cases occurred in the state of Bavaria, south-eastern Germany, however, the region endemic for BoDV-1 with so far only few known human cases extends further north and east to other German federal states [9]. Infected shrews excrete the virus through feces, urine, saliva or skin [1–3] and indirect transmission is likely since explicit transmission events and risk activities could not be identified [10]. Living in rural areas and outdoor or agricultural work were speculated to be risk factors [11]. A recent epidemiological study revealed that very rural living with residences directly abutting fields or natural areas conveys an elevated risk for BoDV-1 disease compared to matched controls in multivariable analyses [10]. BoDV-1 transmission occurs in the peridomestic surroundings according to the same epidemiological study [10] and phylogenetic analyses [11, 12]. Still, the portal of entry for the virus in the human host and the incubation time are unknown. No therapy is established for this nearly uniformly fatal disease. In a recent study on 20 PCR confirmed BoDV-1 encephalitis cases, the median survival time since symptom onset was 5.5 weeks [10].

Here, we describe the detection, clinical course, treatment attempts and epidemiology, as well as the current gaps of knowledge regarding therapy, transmission and prevention of two fatal pediatric cases constituting the first identified local BoDV-1 encephalitis cluster. The cases occurred within a time period of three years in the same small village in South-East Bavaria, Germany.

## Materials and methods

### Case detection, bornavirus serology, and ethical clearance

A validated diagnostic testing scheme for the rapid *intra vitam* diagnosis of human bornavirus encephalitis is used which includes serology and molecular testing, alongside a graded case definition for possible, probable and confirmed BoDV-1 encephalitis [12]. Detected cases are notified to local health authorities immediately.

The serological workflow consists of an indirect immunofluorescence assay (IFAT) with a persistently BoDV-1 infected cell line for serological screening of serum and cerebrospinal fluid (CSF), followed by a line blot assay for confirmation [12]. The line blot utilizes recombinant BoDV-1 phosphoprotein (P) antigen with a cut-off of 16 arbitrary units (AU). Sera and CSF samples of patients with confirmed BoDV-1 encephalitis served as positive controls for both serological assays.

Ethical clearance was granted from the Medical Board of Hamburg (No. PV5616) for this study. Written consent was obtained from the next-of kin of the presented cases. The genetic study for possible immunodeficiency was approved by the institutional review boards of the University Hospital LMU Munich and conducted in accordance with current ethical and legal frameworks.

### RNA extraction and BoDV-1 polymerase chain reaction testing

Ten µm thick sections of formalin-fixed and paraffin-embedded (FFPE) brain tissue from various anatomical regions were used for viral RNA isolation. The RNA was extracted from the FFPE tissue using RecoverAll^™^ Total Nucleic Acid Isolation Kit for FFPE (Life Technologies, Carlsbad, CA, USA), according to the instructions of the manufacturer. Quality and integrity of the extracted RNA was performed using Qubit^™^ RNA HS Assay Kit (Thermo Fisher Scientific, Austin, TX, USA) and Agilent 2100 Bioanalyzer (Agilent, Santa Clara, CA, USA).

Molecular testing for BoDV-1 confirmation was performed by quantitative reverse-transcription real time polymerase chain reaction (qRT-PCR) for BoDV-1 [13] from CSF and FFPE brain tissue sections. BoDV-1 copy numbers were calculated using the standard curve of the titrated synthetic positive control oligonucleotide with known molarity and copy numbers [7].

### BoDV-1 genome sequencing and virus phylogeny

Viral metagenomic sequencing was performed on a NextSeq 2000 platform using the 200-cycle (2 × 100 bp paired-end) NextSeq2000 P2 reagent kit (Illumina, San Diego, CA, USA) according to a protocol described elsewhere [14] from native brain tissue attached to a CSF probe in Case 1 [12] and from the basal ganglia region of Case 2. The raw data generated were cleaned from low-quality reads and polyclonal sequences were removed. The sequence analysis, viral genome assembly, genomic organization, and multiple alignments of BoDV-1 were performed using Geneious Prime (Biomatters, Auckland, New Zealand).

The phylogenetic relationships of the BoDV-1 genomes were analyzed by constructing a maximum likelihood phylogenetic tree using PhyML 3.0 (https://www.atgc-montpellier.fr/phyml/versions.php). The Akaike information criterion was chosen as the model selection framework and the GTR + I + G as the best model.

### Patient whole exome sequencing for immunodeficiency

Peripheral blood from patients and healthy parents were acquired upon written consent and used for genomic DNA extraction (Qiagen, Hilden, Germany). Whole exome sequencing libraries were prepared using Illumina DNA prep reagents (Illumina, San Diego, USA) and TWIST Humane Core + RefSeq (Case 1) or TWIST comprehensive exome (Case 2) enrichment (Twist Bioscience, South San Francisco, USA). Sequencing was performed using an Illumina NextSeq 500 (Case 1) and NovaSeq 6000 (Case 2) sequencing platform. The bioinformatic analysis included standard tools, such as Burrows-Wheeler Aligner (BWA 0.7.15), Genome Analysis ToolKit (GATK 3.6), Variant Effect Predictor (VEP 89) and frequency filters with public and *in-house* databases (e.g., GnomAD). Genetic variants were analyzed in context of known primary immunodeficiency genes [15].

### Bioinformatic modelling for remdesivir and BoDV-1 RNA-dependent RNA polymerase

To assess the potential activity of remdesivir against BoDV-1 RNA-dependent RNA polymerase (RdRP; Uniprot P52639), Modeller 9.22 software was used and molecular docking was tested employing UCSF Chimera 1.14 [16] and AutoDock Vina 1.1.2 [17]. The SARS-CoV-2 RdRP structure bound to remdesivir (PDB ID 7B3B [18]) was used as a template to generate homology models.

### Histology and immunohistochemistry

A brain sample of Case 1 and multiple autoptic brain tissue samples from various anatomical regions of Case 2 were fixed in formalin and embedded in paraffin for routine histology. In addition to H&E, immunohistochemical staining for BoDV-1 P antigen was conducted as described before [19]. Staining against CD3 (polyclonal; Agilent Technologies, Santa Clara, CA, USA) and CD20 (L26; Agilent Technologies) were performed in an automated manner on a VENTANA BenchMark ULTRA slide stainer (Roche Diagnostics, Rotkreuz, Switzerland) using antibody dilutions of 1:50 and 1:100, respectively, and the UltraView Universal DAB Detection Kit (Roche Diagnostics) for detection of the immunohistochemical signal.

### Epidemiology

In detected cases, structured interviews with a semiqualitative questionnaire for possible risk factor elucidation and activities of the patients were conducted among the next-of-kin by at least two epidemiologists. Detailed information on the children´s daily routine, outdoor activities, range of motion including traveling, contact to animals (especially cats and shrews), potential exposure to small mammals´ secretions, contact to water bodies, personal diet and residence was gathered. In addition, bornavirus serology was offered to the relatives.

## Results

Since 2018, 17 human BoDV-1 cases were diagnosed at the Bernhard Nocht Institute, five of them retrospectively. In 2021 and 2022, three and five cases were diagnosed at this institution and notified to local health authorities, respectively. Here, we focus on two pediatric cases treated at the Dr. von Hauner Children’s Hospital, Ludwig-Maximilians- University, Munich (Cases 1 and 2) from the same small village, as these cases form the first detected cluster of BoDV-1 encephalitis and stand further out in terms of age of the patients, diagnosis and treatment.

### Case 1

The first patient, an 11-year-old girl, was transferred to the pediatric intensive care unit (PICU) of the Dr. von Hauner Children’s Hospital in Munich from a peripheral hospital in Bavaria with suspected autoimmune encephalitis in autumn 2019. The detection of the case during the ongoing prospective study has been reported elsewhere [12]; in this report here, the full clinical picture and the course are described, especially in comparison to Case 2.

**Fig. 1:**
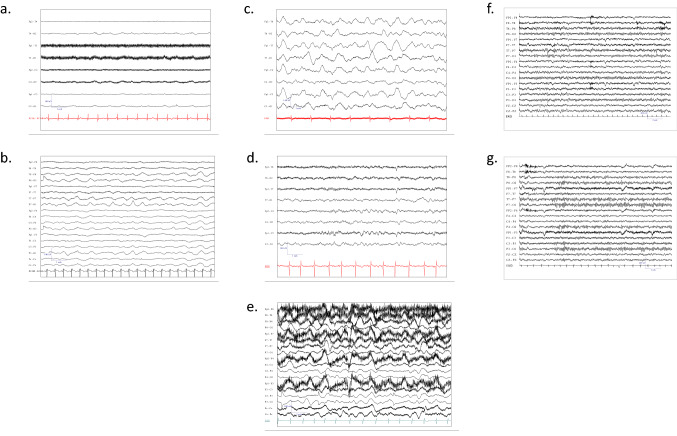
Electroencephalograms of the two cases during the course of disease and healthy controls. Electroencephalogram recording of Case 1 on day 20 of disease (admission to the LMU intensive care unit) showing a general suppression pattern (wave frequency of 2–3/sec (Hertz)) (**a**), and last recording demonstrating slowing and deterioration of the suppression pattern (1–2 Hertz) (**b**). Early recording of Case 2 on day 13 showing continuous slowing (3–4 Hertz) (**c**), temporal improvement with sleep spindles on day 22 (**d**), and finally on day 46 with delta coma (1–2 Hertz delta waves) (**e**). Six-year-old healthy matched control awake electroencephalogram recording with normal 7–8 Hertz activity (**f**). Eleven-year-old matched control awake electroencephalogram recording with normal 8–9 Hertz activity (**g**). Bottom line represents electrocardiogram (ECG)

**Fig. 2:**
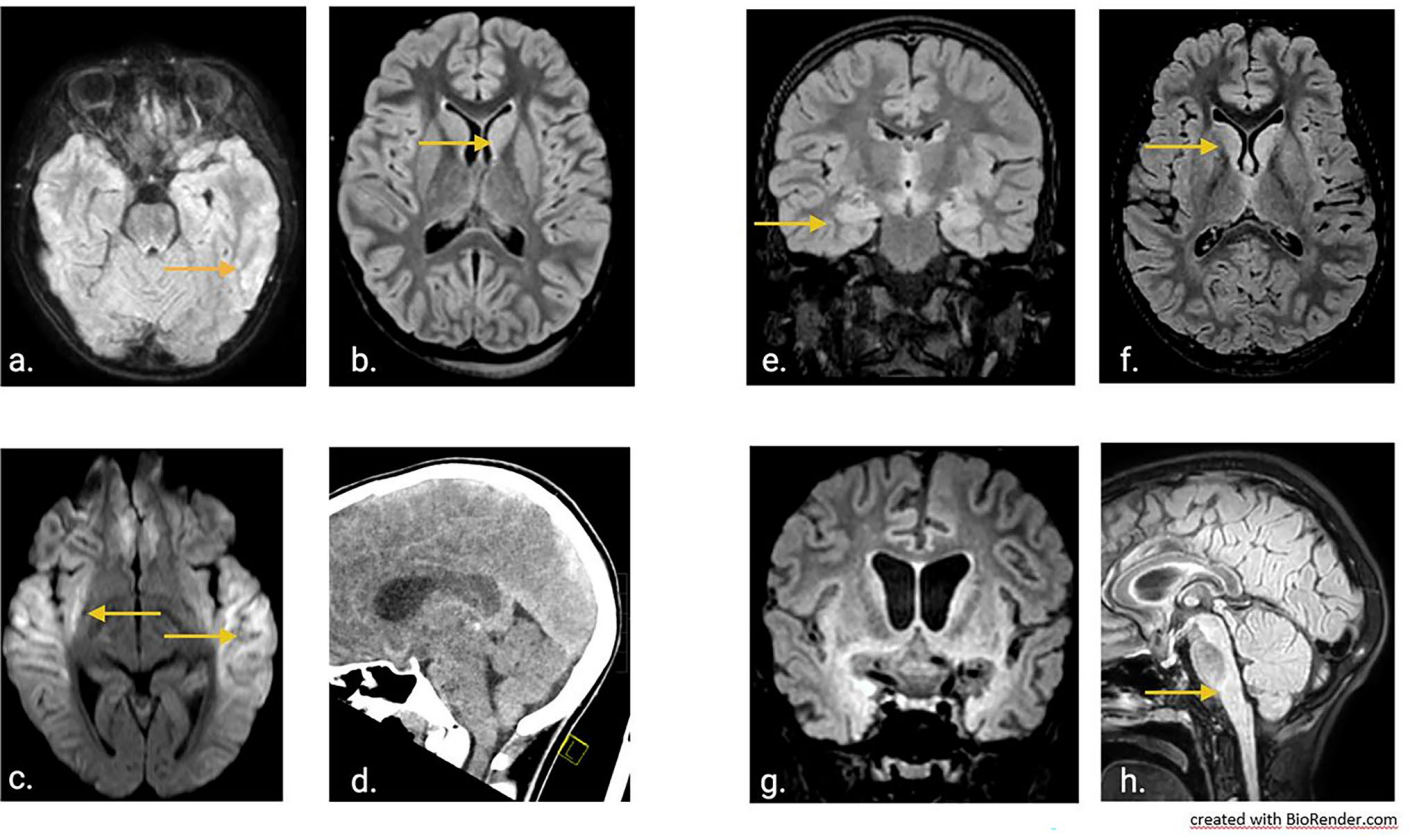
Brain magnetic resonance imaging of the two pediatric cases over time. Initial scans of Case 1 on day 8 after symptom onset showing signal hyperintensity in the parietal brain regions (fluid-attenuated inversion recovery (FLAIR) sequence) (**a**) and in the basal ganglia on the left on day 12 (FLAIR sequence) (**b**). Later scans of the same case on day 15 demonstrating signs of cytotoxic edema (diffusion weighted imaging) (**c**), and last computed tomography scan on day 29 with diffuse edema (**d**). Initial scans of Case 2 on day 12 of disease showing signal alterations in temporal brain regions (FLAIR sequence) (**e**) and in the basal ganglia (FLAIR sequence) (**f**). Late scans on day 51 demonstrating brain atrophy and signal hyperintensity in the basal ganglia and temporomesial (FLAIR sequence) (**g**), as well as in the brain stem (FLAIR sequence) (**h**)

The girl had presented initially to a peripheral hospital with fever, headache, severe malaise for one week and a presumed first epileptic seizure. There had been normal development and absence of medical preconditions. After initial admission in a stable general condition, a rapid decline of vigilance and several seizures occurred. An electroencephalogram (EEG) had shown diffuse slowing (Fig. 1a). Cranial magnetic resonance imaging (MRI) had shown signal alterations in the parietal brain regions on both sides and in the basal ganglia on the left side (Fig. 2 a, b). Lumbar puncture had demonstrated slight elevation of cells. As an infectious etiology was suspected, the girl was treated with acyclovir and doxycycline. Five days after admission, the girl had to be intubated and mechanically ventilated as pupils were fixed and vigilance was persistently decreased. A cranial computed tomography (CT) had shown no evidence of increased intracranial pressure and follow-up MRI had demonstrated a slight improvement with fewer signal alterations. A subsequent extubation attempt had failed, however. The next MRI scan, 4 days later, had shown signs of cytotoxic edema (Fig. 2c). The EEG still demonstrated a diffuse slowing/suppression pattern, consistent with encephalopathy (Fig. 1b). No infectious agent was detected and in the course of the disease, dexamethasone (0.4 mg/kg/d) for 4 days (from day 10 to 14) and afterwards methylprednisolone (20 mg/kg/day) for 5 days (from day 14 to 19) had been given, as well as intravenous immunoglobulins (0.4 g /day) for 5 days (from day 14 to 19), as an autoimmune pathogenesis had been assumed alternatively. During therapy, the patient’s condition had however further deteriorated.

**Fig. 3:**
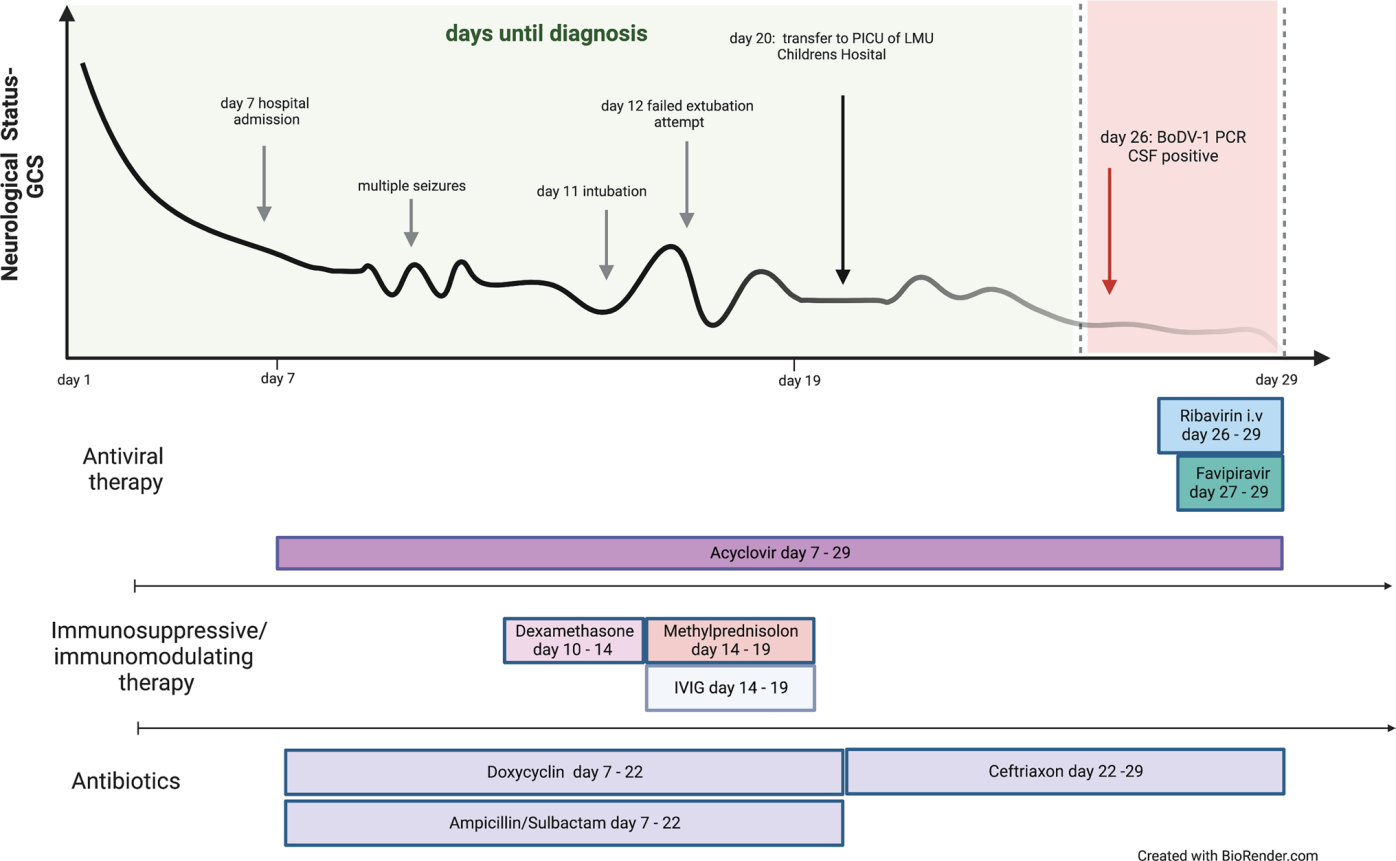
Clinical course and treatment overview of Case 1. The figure shows the clinical development during the 29-day-long course of disease, the time elapse until diagnosis, as well as antiviral, immunosuppressive and antibiotic therapy. GCS, Glasgow Coma Scale. IVIG, intravenous immunoglobulins

The patient was thereupon transferred to the Dr. von Hauner Children’s Hospital with suspected autoimmune encephalitis after 20 days of disease for possible plasmapheresis. However, no autoantibodies were detected and more extensive testing for infectious agents was initiated, including for BoDV-1. Testing for bornavirus-reactive antibodies was positive in serum and CSF when tested on day 26 with IFAT titers of 2560 and 160, respectively. Line blot results for antibodies against BoDV-1 P in serum and CSF were 17 and 2 units, respectively. A qRT-PCR for BoDV-1 was positive from CSF (cycle threshold (ct) value of 33.0) on the same day, and BoDV-1 encephalitis was diagnosed. Antiviral therapy was initiated with ribavirin (loading dose 30 mg/kg/day; on day 2 and following days: 64 mg/kg/day in 4 doses), and, during the course, favipiravir was added (day 1: 1600–1600–800 mg; day 2 and following days: 800 mg bid; body weight 40 kg). Clinically, the girl showed refractory increased intracranial pressure during the course. With clinical and imaging morphologic signs of beginning cerebral herniation due to generalized cerebral edema (Fig. 2d), the girl died on day 29 of disease and four days after the diagnosis of BoDV-1 encephalitis. Genetic testing by whole exome sequencing showed no hits for an immunodeficiency. The clinical course and the respective treatment is shown in Fig. 3.

**Fig. 4:**
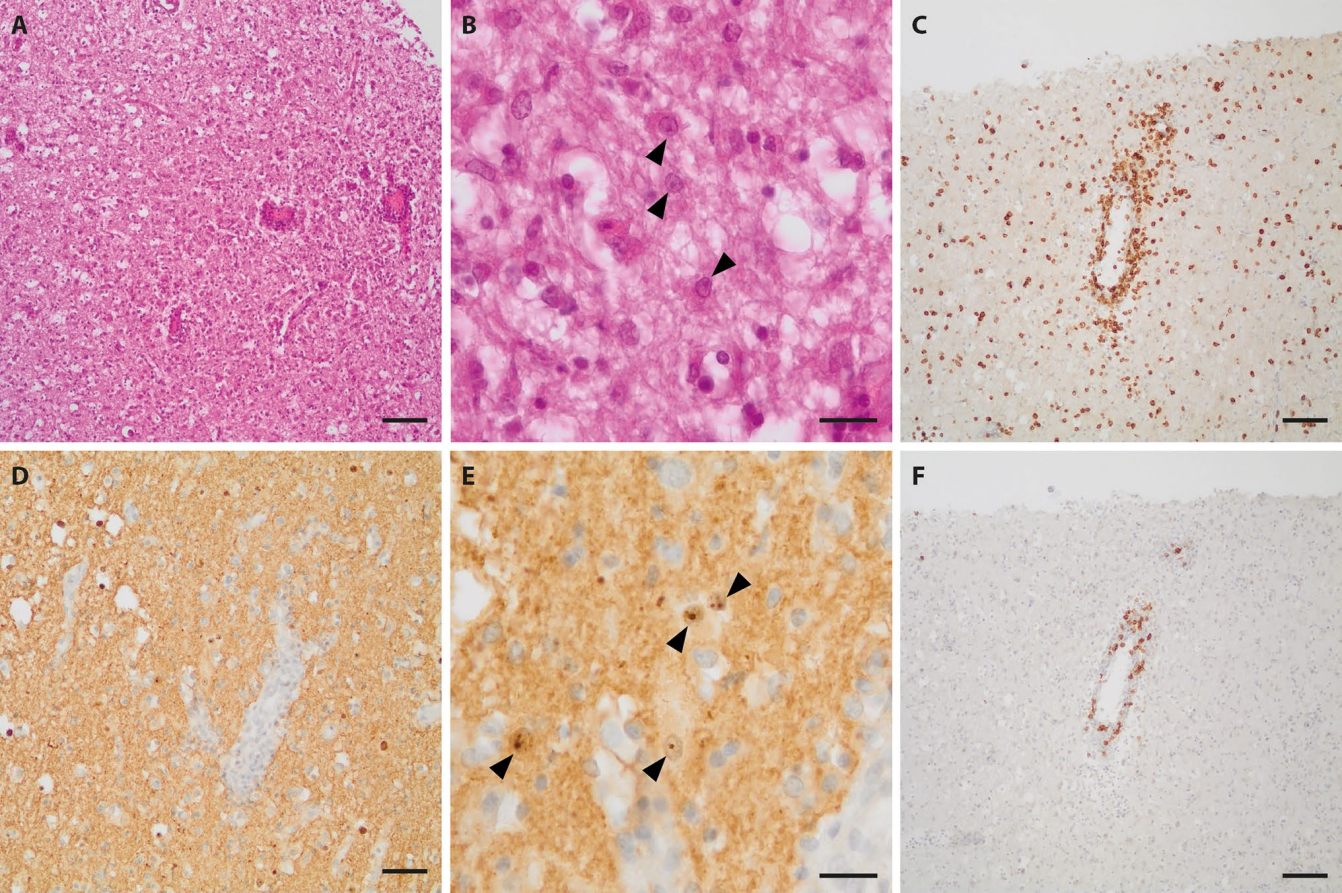
Histology and immunohistochemical staining of brain from Case 1. On H&E, severe parenchymal destruction with almost complete neuronal loss, prominent reactive astrogliosis with enlarged reactive astrocytes with intranuclear viral inclusions (bornavirus-specific Joest-Degen bodies; arrowheads) and diffuse and perivascular accentuated inflammatory infiltration could be observed (**A**,** B**). Immunohistochemical staining for BoDV-1 P antigen demonstrated strong diffuse staining with labeling of intranuclear viral inclusions (**D**,** E** arrowheads). While infiltrates of CD3-positive T lymphocytes showed a diffuse as well as perivascular accentuated pattern, CD20-positive B lymphocytes were in total less frequent and primarily arranged in perivascular cuffs (**C**,** F**). Scale bars: **A**, **C**, **D**, **F**: 100 µm; **B**, **E**: 10 µm

No autopsy was performed, but histological examination of a brain tissue sample obtained on day 29 revealed severe destruction of the brain parenchyma with nearly complete neuronal loss and massive reactive astrogliosis. Reactive astrocytes often beared small eosinophilic intranuclear viral inclusions. A diffuse as well as perivascular accentuated inflammatory infiltration reflecting sclerosing lymphocytic encephalitis was seen (Fig. 4A, B). Immunohistochemistry for BoDV-1 P antigen showed largely diffuse staining of the tissue with occasional strong labeling of single nuclei and particularly nuclear inclusion bodies (Fig. 4D, E). The inflammatory infiltrate was mainly composed of CD3-positive disseminated as well as perivascular accentuated T lymphocytes, to a lesser extent of predominantly perivascular CD20-positive B lymphocytes (Fig. 4C, F).

The complete BoDV-1 genome was successfully reconstructed from the brain tissue sample of this patient previously (GenBank accession no. MT364324; ([12]).

### Case 2

The patient, a previously healthy 6-year-old boy, was transferred to the PICU of the Dr. von Hauner Children’s Hospital in early summer 2022 from the same peripheral hospital in Bavaria, about 3 years after Case 1. The patient had initially shown high fever, headaches, and intermittent vomiting, followed by disorientation. On day 9 of disease, he had been admitted to his local hospital. An EEG had shown diffuse slowing (Fig. 1c). MRI of the brain revealed discrete signal alterations in the temporal brain regions and basal ganglia (Fig. 2e, f). Lumbar puncture had demonstrated a moderate mono-lymphocytic pleocytosis (118 cells/µl; normal < 5), a slight elevation of protein (46.8 mg/dl; normal 15–45), with normal lactate (1.6 mmol/l; normal 1.1–2.4) and glucose levels (61 mg/dl; normal 60–80). He was started on empiric antibiotic therapy (ampicillin, cefotaxime and clarithromycin) as well as on acyclovir for a presumed infectious cause.

The treating physicians were well aware of Case 1 and requested immediate testing for BoDV-1. On day 12, bornavirus serology was negative from blood and CSF, but BoDV-1 qPCR was positive from CSF (ct 32.8) and BoDV-1 encephalitis was diagnosed. As the patient’s status deteriorated, he was transferred to the PICU of the Dr. von Hauner Children’s Hospital on day 12; on the same day seroconversion in the IFAT only was demonstrated (1:20 in serum; negative in CSF). The patient required immediate mechanical ventilation due to severely reduced conscious level (Glasgow Coma Scale (GCS) 5–7). An intensified treatment attempt was initiated immediately. He was started on an antiviral combination including enteral administered favipiravir (day 1: 1200 mg-1200 mg-600 mg, following days until day 55: 600 mg bid (body weight ca. 23 kg)), ribavirin as intravenous administration (loading dose 33 mg/kg/day, following days 60 mg/kg/day in 3 single doses, reduction to 30 mg/kg/day due to hemolytic anemia on day 23) and intrathecal injection (day 1: 1 mg/kg/day, days 2–4: 2 mg/kg/day, days 5–8: 3 mg/kg/day). After bioinformatically calculated docking scores for remdesivir and BoDV-1 RdRP showed values of -7.9 (for SARS-CoV-2 RdRP: − 6.5), a block of viral replication appeared plausible as lower scores indicate tighter binding. Thereupon, remdesivir (loading dose 5 mg/kg/day, following days 2.5 mg/kg/day in one single dose) was added. Moreover, different immunosuppressants including high-dose steroids (20 mg/kg/day for 5 days), ciclosporin A (starting with 4.3 mg/kg/day in two single doses, targeted plasma level 80–100 ng/ml)) and mycophenolate (50 mg/kg/day in two single doses) were given. On day 14 BoDV-1 qPCR from CSF showed ct values of 33.9 and on day 17, qPCR was negative from CSF, serum, full blood, urine, stool, and saliva. His condition started to improve and he was successfully extubated on day 21. In the following days the antiviral treatment with ribavirin was reduced due to side effects of the drugs (hemolytic anemia) and was stopped on day 31. Remdesivir was stopped on day 25, because the normally recommended treatment duration in other indications (e.g., COVID-19) is 10 days. The patient had preserved long-term memory and intermittently compromised short-term memory. He was able to read his comic books and sit in a wheelchair. The EEG results had shown improvement as well (Fig. 1d).

**Fig. 5:**
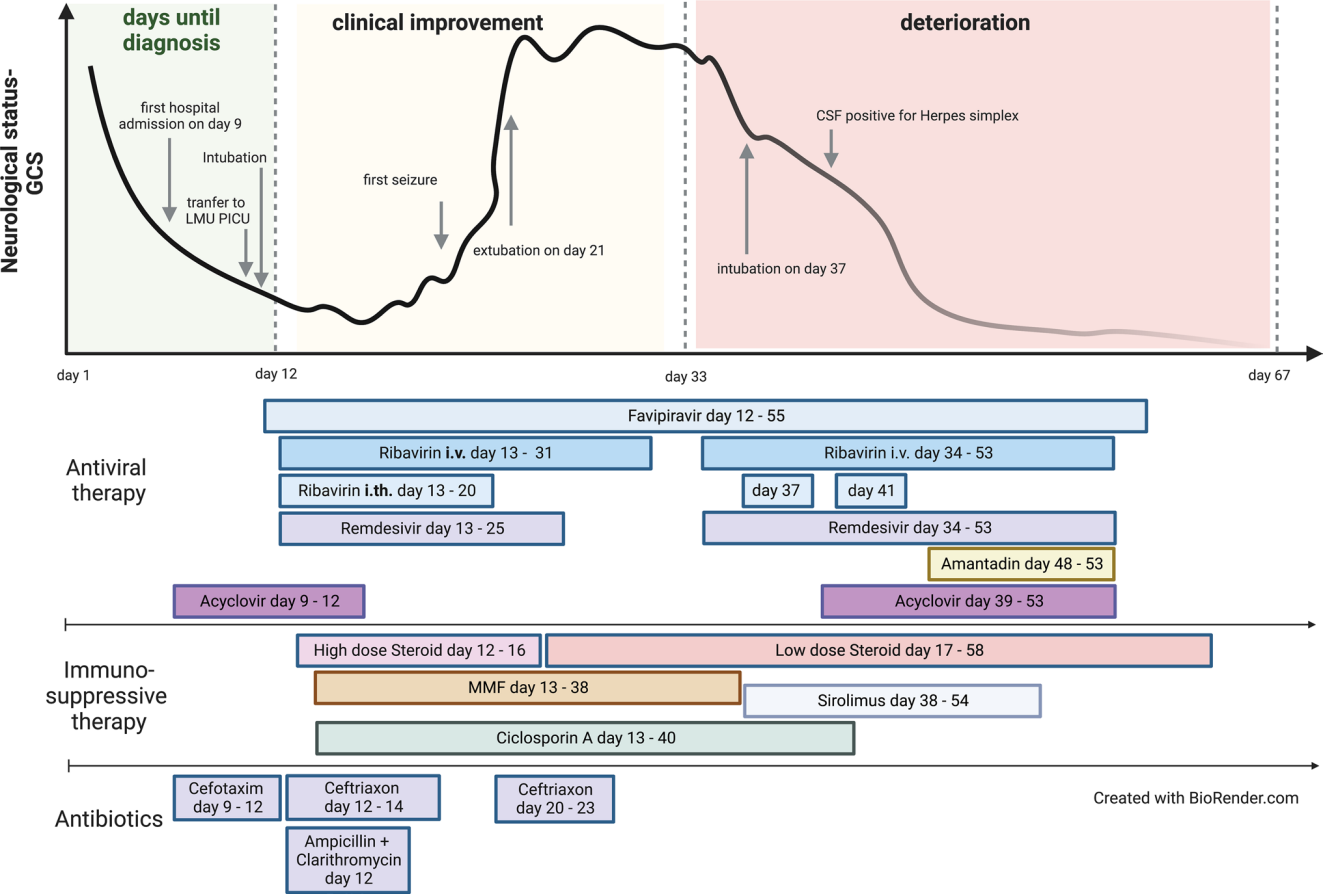
Clinical course and treatment overview of Case 2. The figure demonstrates the prolonged clinical course of 67 days, the time elapse until diagnosis, as well as the intensified antiviral and immunosuppressive therapy, and the initial antibiotic treatment. GCS, Glasgow Coma Scale

However, on day 31, BoDV-1 qPCR was positive in CSF (ct 38.8) and serum antibody levels were 1:320 by IFAT. On day 33, a progressive vigilance decline was noticed and the patient struggled to cough effectively. MMF was switched to sirolimus (0.2 mg/kg/day in one single dose) on day 38 to target T-cells more effectively, and remdesivir and ribavirin were started again on day 34. On day 37, he had to be re-intubated and mechanically ventilated again. The EEG showed delta coma (Fig. 1e). On day 41 HSV-1 DNA was detected in CSF by PCR and on the skin of the face, and acyclovir was again administered. Ciclosporin A was stopped on day 41 to reduce immunosuppression. BoDV-1 qPCR was positive in CSF (ct 34.2), but negative in tear fluid, stool, tracheal secretion and serum. On day 48 amantadine (5 mg/kg/day in 2 single doses) was given additionally. Three days later, BoDV-1 RNA was detected in saliva (ct 35.7) and tear fluid (ct 35.4), as well as in CSF (ct 33.8) by qPCR, but not in stool or urine; the patient was thereupon isolated and barrier nursing was performed although virus isolation attempts on Vero and CRFK cells were negative. Serum IFAT was 1:1280 with a positive line blot result for BoDV-1 P antigen with 24 units. In the following days he presented in a minimal conscious state with decerebrate rigidity. No clinical improvement was observed, and MRI demonstrated brain atrophy and signal hyperintensity in the basal ganglia, temporomesial and in the brain stem one day later (Fig. 2g, h). The patient died on day 67. Genetic testing by whole exome sequencing had shown no signs of immunodeficiency in this patient either. An overview of the clinical course and treatment is shown in Fig. 5.

**Fig. 6:**
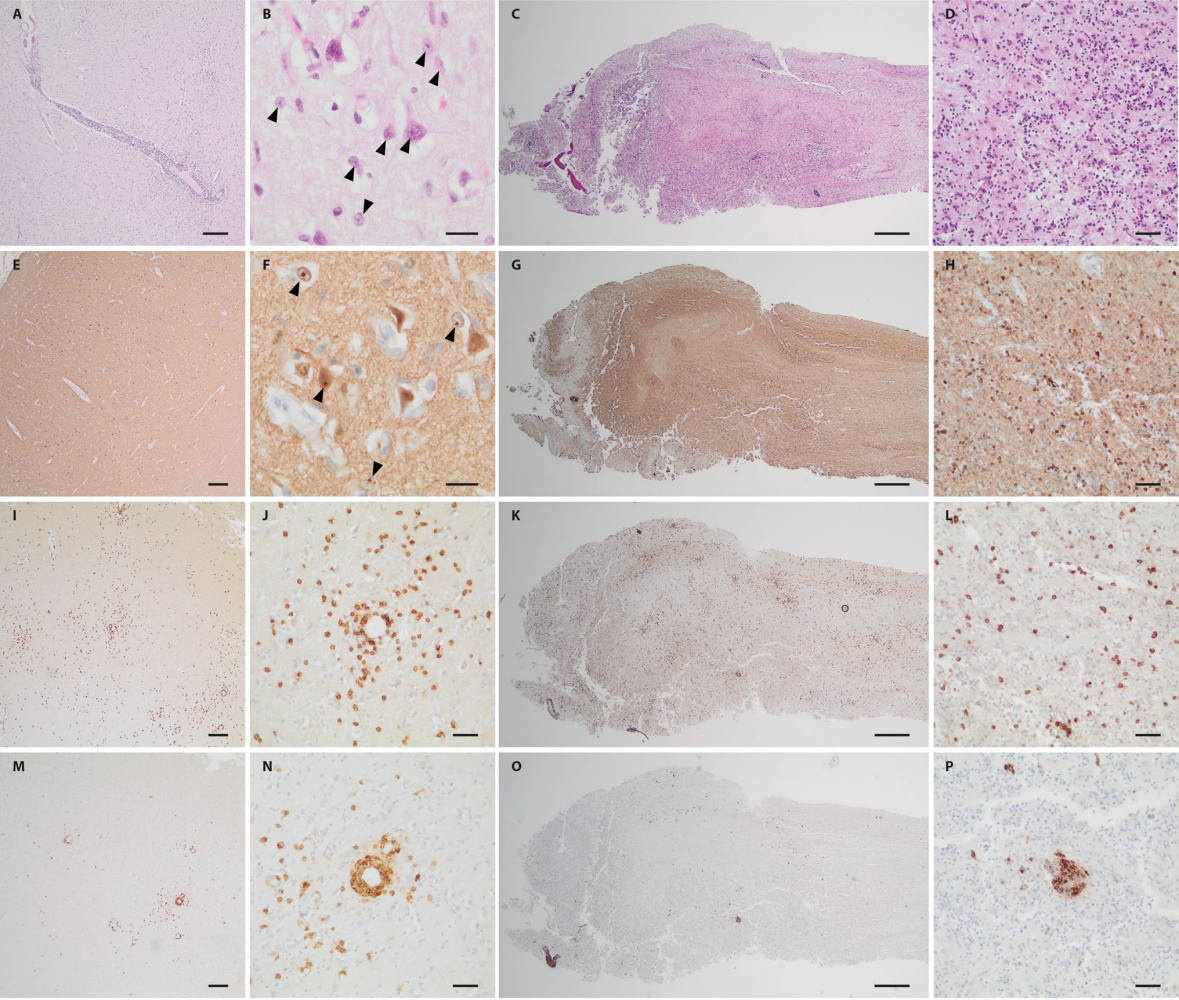
Histology and immunohistochemical staining of brain and olfactory nerve from Case 2. H&E stained sections of the different brain regions sampled (shown here: cingulate gyrus) demonstrated variably pronounced destructive changes with neuronal loss, reactive astrogliosis and inflammatory infiltration (**A**,** B**). Joest-Degen nuclear inclusion bodies (arrowheads) could be detected on higher magnification in neurons and astrocytes (**B**). On immunohistochemistry, strong diffuse positivity for BoDV-1 P antigen including intranuclear Joest-Degen bodies (arrowheads) could be observed (**E**,** F**). While CD3-positive T-lymphocytes showed some perivascular accentuation, but were also rather disseminated (**I**,** J**), CD20-positive B lymphocytes were arranged in perivascular cuffs (**M**,** N**). Similar changes were encountered in the olfactory bulb and tract (respective opposite panels). Stainings** C**, **D**: H&E; **G**, **H**: BoDV-1-P; K,I: CD3; **O**, **P**: CD20. Scale bars: **E**, **I**, **M**: 200 µm; **A**, **C**, **G**, **K**, **O**; 100 µm; **D**, **H**, **J**, **L**, **N**, **P**: 50 µm; **B**, **F**: 10 µm

Neuropathological examination of the autopsied brain showed a sclerosing lymphocytic panencephalitis characterized by variably pronounced neuronal loss and reactive astrogliosis with enlarged reactive astrocytes and perivascular and diffuse lymphocytic infiltration (Fig. 6A, B). Eosinophilic intranuclear viral inclusions were apparent in reactive astrocytes as well as in neurons (Fig. 6B). Similar changes could be observed in the olfactory bulb and tract (Fig. 6C, D). Strong immunohistochemical positivity for BoDV-1 P antigen was demonstrated in all brain regions including the olfactory bulb and tract (Fig. 6E–H). In contrast, immunohistochemical staining for herpes simplex virus was negative (not shown). Immunohistochemistry for CD3 (Fig. 6I–L) and CD20 (Fig. 6M–P) revealed a perivascular as well as disseminated pattern for CD3-positive T lymphocytes, whereas CD20-positive B lymphocytes were primarily perivascular arranged. Peripheral tissues were not available for examination.

Quantitative BoDV-1 PCR results showed highest viral concentrations in structures of the limbic system (cingulate gyrus and amygdala), the olfactory bulb and tract, the thalamus, superior frontal and medial temporal gyri, and the inferior parietal lobule (Table 1). In contrast, HSV-1 PCR was negative from the brain tissue sections. The complete BoDV-1 genome was successfully recovered from the basal ganglia region with a median sequencing depth of 1337x (GenBank accession no. OP776335).

**Table 1:** BoDV-1 copy numbers in different brain regions and cranial nerves

Brain region / cranial nerve	PCR ct value	BoDV-1 copies/ng RNA
Superior frontal gyrus	17.94	194,444
Olfactory bulb and tract (left)	18.83	192,593
Thalamus	19.73	180,702
Amygdala	17.33	175,439
Superior and medial temporal gyrus	18.89	166,970
Inferior parietal lobule	19.66	118,787
Anterior cingulate gyrus	19.97	102,534
Precentral gyrus	19.76	88,596
Trigeminal nerve (left)	20.88	87,361
Anterior striatum	20.2	81,603
Anterior hippocampus	19.96	76,149
Lower medulla oblongata	20.73	74,180
Lower pons	21.05	67,531
Medial frontal gyrus	19.82	65,986
Facial nerves (left + right)	20.99	65,077
Upper medulla oblongata	20.53	61,856
Midbrain	20.52	60,333
Upper pons	21.69	58,442
Basal ganglia at the level of the mammillary bodies	19.94	54,806
Trigeminal nerve (right)	21.59	35,667
Posterior hippocampus	22.23	29,206
Striate area	20.81	27,407
Cerebellar hemisphere	21.96	22,625
Choroid plexus	23.74	19,900
Pineal gland (Epiphysis)	23.56	17,239
Cerebellar vermis	21.64	14,342
Oculomotor nerve (right)	24.87	8392
Vestibulocochlear nerve (right)	24.29	7915
Abducens nerve (right)	25.9	3974
Optic nerve (right)	25.81	3195
Optic nerve (left)	24.13	2148
Oculomotor nerve (left)	26.6	1492
Adenohypophysis	26.06	417

Not all cranial nerves could be recovered during autopsy. Regions analyzed are sampled according to a standardized brain autopsy scheme. *Ct* cycle threshold

### BoDV-1 phylogeny

**Fig. 7:**
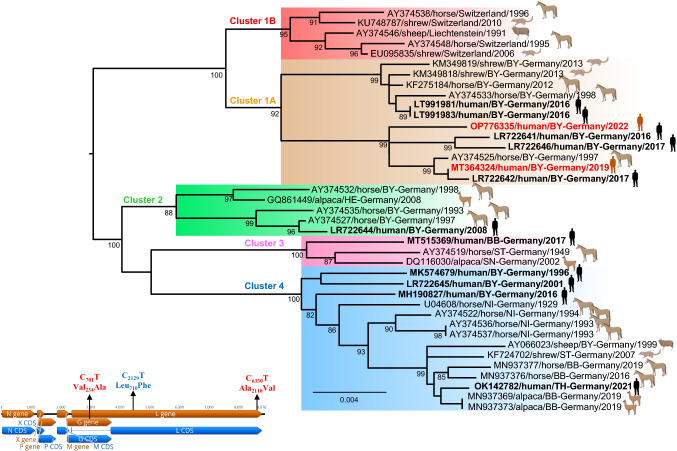
Maximum likelihood phylogenetic tree of the BoDV-1 strains from the two pediatric patients, based on partial N and P gene nucleotide sequences (~ 1824 bp), as well as depiction of unique mutations of the genome of both strains. Maximum-likelihood bootstrap replicate scores (> 70%) are shown next to the nodes. Taxon information includes GenBank accession number, host species, country of origin (for Germany—federal states (BY-Bavaria; HE-Hesse; BB-Brandenburg; ST-Saxony-Anhalt; SN-Saxony; NI-Lower Saxony; TH-Thuringia) and year of detection are provided. BoDV-1 sequences obtained from the two patients in this study are shown in red. BoDV-1 sequences from humans are depicted in bold. Color code of the clusters are according to [11, 12]. The scale bar indicates nucleotide substitutions per site. The left lower inset shows unique non-synonymous mutation observed in the BoDV-1 genome from the patient OP776335 (mutations in red) and patient MT364324 (mutation in blue)

Phylogenetic reconstruction showed that the BoDV-1 sequences recovered from the two patients of this study belong to cluster 1A, which also encompasses virus sequences from infected shrews, horses, and further human patients in Bavaria, southern Germany. Moreover, although the strain OP776335 originates from the same village as strain MT364324 detected in 2019, they do not cluster together in the phylogenetic tree, thus representing different BoDV-1 variants (Fig. 7).

The nucleotide sequence similarity with the close relatives from the cluster 1A ranged from 97.2 to 99.1% of the whole BoDV-1 genome, whereas amino acid sequence conservation ranged from 99.6 to 99.8%. Comparison of the genomic sequences between both cases as well as with the other BoDV-1 strains showed several unique synonymous substitutions along the genomes with two unique amino acid substitutions in the G and L protein in the strain OP776335 and one in the L protein of the strain MT364324 (Fig. 7, left lower inset).

### Epidemiology

Both children had lived with their families in the same small village with roughly 2000 inhabitants in the rural countryside of South-East Bavaria. To our knowledge, there had never been a local case cluster before within this geographical proximity in the entire group of more than 40 cases (since 1992) notified to the Robert Koch Institute. The family houses, both detached houses with gardens, were located on opposite fringes of the village next to open agricultural fields or amidst fields, respectively, with an air-line distance of around 750 m. Both children had lived in the village their whole life, attended kindergarten there and the older child later went to school in the neighboring town. They spent most of their time in the surroundings of their family homes, playing with other children in the gardens, playgrounds, forests, or in the village. Both children enjoyed gardening with their families, one child practiced track and field outdoors, the other played outdoor soccer in the local sports club. Since the families were living on opposite sides of the village and the children did not have the same age, they did not know each other personally and did not have the same friends. Neither family could imagine a common source of infection. One family held cats and rabbits while the other did not have any domestic or farming animals and cared for the neighbors’ chicken and dog only. Other regular close contacts to animals included cattle in one case. Besides that, regular close contacts to animals were denied. Members of both families confirmed shrew presence on the premises but could not confirm direct shrew contact or name a plausible transmission event. Both families assumed their child would not deliberately touch a shrew or a shrew’s carcass. Other commonalities than the ones mentioned could not be found in the two cases.

Bornavirus serology of the next of kin (four household members of the two families, respectively) was negative.

## Discussion

We here describe the first detection of a geographical cluster of BoDV-1 encephalitis and report the clinical work-up, treatment attempts and epidemiological considerations of this rare but nearly always fatal emerging disease. While some aspects of the two cases analyzed here in-depth are typical for what is currently known about the disease (MRI changes, neuropathology findings), the two cases stand out among other human BoDV-1 encephalitis cases regarding the age of the patients, the rapidity of diagnosis and initiation of treatment. For the first time, we report a clinical improvement during human BoDV-1 encephalitis and not merely a stabilization, when off-label therapy was commenced early. In addition, we here provide evidence for the portal of entry of BoDV-1 into the human host. Moreover, we show for the first time excretion, at least of viral RNA, by the human host.

### Antiviral and immunosuppressive treatment considerations

Though rapid diagnosis is the basis for therapy, no treatment regimen is established for human BoDV-1 encephalitis or has been shown to be effective except for two cases left severely disabled [4, 20]. Moreover, so far no therapeutic antibodies have been developed and a vaccine is not available. The circumstances that bornaviruses establish persistent infections intranuclearly by attachment to chromosomes [21] and that tissue destruction is caused by the host’s immune response as shown in animals [6] led to the conclusion that a combined antiviral and immunosuppressive therapy might be the best approach, ever more, as recently severe tissue destruction [8] and pro-inflammatory immunodysregulation [7] has been demonstrated in humans as well. However, the dilemma of extended, possibly life-long treatment (owing to the natural history of the virus and the disease) with secondary complications arising thereof becomes apparent and a complete elimination of a persistent virus from already infected brain cells does not seem to be realistic.

In vitro experiments with favipiravir (T-705; 6-fluoro-3-hydroxypyrazine-2-carboxamide), a synthetic guanidine nucleobase licensed for influenza treatment in Japan, and ribavirin (1-β-D-ribofuranosyl-1,2,4-triazol-3-carboxamide), a long-known guanosine analog, have shown antiviral effects against BoDV-1 [22, 23]. RNA virus replication is inhibited by both drugs, targeting viral polymerases and suppressing viral RNA levels. Favipiravir acts more efficiently [22]. There are no published results from in vivo studies for favipiravir yet. Studies examining the effect of favipiravir in animals infected with rabies virus, a virus from the same mononegavirales order like BoDV-1, showed antiviral effects however [24]. However, neither the dose or duration for BoDV-1 treatment nor detailed pharmacokinetics (CSF or brain parenchyma penetration) or interactions are known to date of this drug. In a few centers in Germany, therapeutic drug monitoring of favipiravir for other purposes is currently set up and will be expanded for bornavirus encephalitis. The compound is available via tertiary hospital pharmacies in Germany upon request via the STAKOB office (Permanent Working Group of Competence and Treatment Centers for High Consequence Infectious Diseases (STAKOB), http://www.rki.de/stakob). Therapy is experimental and side effects are not well studied. Elevated liver enzymes, gastrointestinal, hepatic and hematological side effects have been described.

For ribavirin, data exist from subacute sclerotizing panencephalitis patients showing therapeutic concentrations in the CSF after intravenous administration [25]. Level measurements were not possible in Case 2 due to technical limitations with no laboratory at hand performing the test. Studies in rats with BoDV-1 encephalitis showed clinical improvement and reduction in CD4 and CD8 infiltration of the brain after intrathecal administration of the compound, although the virus concentration did not decrease [23]. Ribavirin is known to cause hemolytic anemia as one of the most prominent side effects.

The rationale for including remdesivir as an additional antiviral agent was based on its known broad-spectrum activity against RNA viruses by targeting a conserved RdRP. Members of the mononegavirales, such as bornaviruses and filoviruses [26], and also the SARS-CoV-2 coronavirus [18], share a negative-strand RNA viral genome that depends on this virally encoded enzyme for replication. As the best docking scores indicated that remdesivir will tightly bind to BoDV-1 RdRP, a block of viral replication seemed plausible. Thus, given the fatal prognosis of BoDV-1 encephalitis and since vast experience on pediatric dosing and tolerability for remdesivir had been gathered during the COVID-19 pandemic, the decision was made to include remdesivir in the antiviral treatment regimen. In addition, amantadine (1-aminoadamantane) was given in a salvage attempt without effect; the efficacy in BoDV-1 infection is mostly seen negative [27]. If any of the antiviral drugs had an effect is hard to analyze as there is no good parameter to measure treatment success or progressive viral spread. It remains unclear whether fluctuating PCR positivity in CSF reflects changing antiviral drug action, as PCR from CSF generally does not seem to be a sufficient tool to monitor therapy success. BoDV-1 does not appear be shed continuously into CSF even during untreated human BoDV-1 encephalitis [28], and the low amount of RNA copies in CSF seen in several studies [11, 12] likely reflects the strong cell-bound nature of the virus. Moreover, an impaired CSF flow from brain to lumbar arachnoid space during the disease could further contribute to the limited sensitivity of BoDV-1 PCR from CSF obtained by lumbar puncture.

Indications that iatrogenically immunosuppressed BoDV-1 encephalitis patients live longer than other BoDV-1 patients became apparent recently [4, 11, 20]. Still, the prognosis is very poor. Of note, initial clinical improvement was noticed in Case 2 to a rapidity and extent never reported before for BoDV-1 encephalitis. However, one 18-year-old patient [20] showed improvement under antiviral and immunosuppressive combination therapy. He has been surviving for nearly one year now, at the cost of a severe mental and physical handicap (unpublished data). Thus, for younger patients, a higher chance for survival might exist. However, neither the particular compound (nor possible combinations), nor the dose and time point to administer immunosuppressive therapy is known or subject of ongoing studies. Any possible individually predisposing factors (if at all)—be it susceptibility to the virus or a higher chance for survival—are so far completely unknown. Whole exome analysis in the two patients presented here revealed no hints for underlying immunodeficiency, including common or known diseases associated with hyperinflammation. EBLN1 and 2 genes were analyzed as well and showed only common single nucleotide polymorphisms.

Immunosuppressive therapy also holds the risk of major complications, especially in an ICU setting, with opportunistic pathogens or reactivation of latent infections. In Case 2 HSV-1 DNA was detected in CSF by PCR, a finding that could not be reproduced by immunohistochemistry or PCR from FFPE tissue of respective temporal brain regions however. Still, reactivation of HSV-1 in the brain and thus a superimposed severe infection would worsen prognosis even further. Whether the immunosuppression delayed seroconversion and/or facilitated the spread of BoDV-1 and shedding (at least of viral RNA) in tear fluid and saliva remains unclear. In animal studies with cyclosporine A-treated rats, immunosuppression was associated with a loss of neurotropism and an atypical dissemination of BoDV-1 [29] and there is thus the potential risk of immunosuppressant-induced viral shedding. Unfortunately, peripheral tissues were not available for analysis in our study and a non-neuronal spread could not be assessed. However, viral RNA was repeatedly not detected in stool and urine in Case 2.

The prolonged course of Case 2 and initial clinical improvement suggest that some of the drugs might have had an effect, but speculation is difficult in this intensive treatment regime with multiple drugs and the lack of established treatment options for BoDV-1 infections. Corticosteroids are important immunosuppressive drugs, and Cases 1 and 2 both received steroids. Case 1 initially received a lower dose and died of elevated intracranial pressure, so it might be speculated that the high-dose steroids in Case 2 had prevented such a complication, resulting in a prolonged course. Case 2 received extensive immunosuppressive medication. Only once in the course of the disease, 3 days before secondary deterioration, the ciclosporin A plasma level was below the target level. If this has had any influence on the course of the disease remains unfortunately unclear. How exactly the mechanism of action of immunosuppressive drugs may aid in the elucidation of the pathophysiology of BoDV-1 encephalitis is of interest. The role of T cells is not clear, though these cells excessively infiltrate the brain tissue [8]; however, bizarre astrocyte changes and enlargement [5, 7, 8] as well as cytokine production by astrocytes as recently shown in human infection [7] strongly point toward astrocyte dysfunction during disease. Toxic glutamate and thus metabolic effects during animal BoDV-1 infection have also been described [6]. Thus, potentially reversible symptoms during the early disease period may result from these dysfunctions and might be pharmacologically targeted. Interestingly, for glucocorticoids a protective role against glutamate toxicity has been demonstrated in neurons [30, 31] and this effect might have led to the initial improvement of Case 2.

### Viral load, BoDV-1 RNA excretion, and possible portal of entry of BoDV-1

As autopsy was only granted in Case 2, viral load distribution in the brain and cranial nerves could only be performed in one of the two patients. As described before [7] high viral loads were seen in inner brain structures, such as the limbic system, thalamus, and others. BoDV-1 RNA was also detected in saliva and tear fluid in Case 2 described here, and thus serous excreta from glands connected to, and controlled by, cranial nerves (glossopharyngeal nerve (which was not available) for saliva and the intermediate part of the facial nerve (which was PCR positive) for saliva and tears). As both nerves have their parasympathetic nuclei in the caudal pons which harbored roughly similar viral loads, centrifugal spread of BoDV-1 from the brainstem via cranial nerves to the glands is highly likely. Of note, all cranial nerves recovered were positive for BoDV-1 RNA, and we recently detected BoDV-1 RNA in tear fluid also of an adult case with marked cranial nerve involvement seen on MRI (unpublished data). Moreover, in a non-human primate model of BoDV-1 encephalitis, BoDV-1 RNA was found in oral swabs of 3/6 monkeys after intracerebral or peripheral inoculation lately [32]. Even though transmission to medical or laboratory personnel has not been described so far [33] and BoDV-1 culture was negative from the patient’s tears and saliva, isolation and hygiene measures should be considered in BoDV-1 infected patients with virus RNA detection in body fluids. Viral culture from materials with high ct values may be futile and may not necessarily exclude infectivity.

Interestingly, the olfactory nerve (bulb and tract) of Case 2 harbored very high viral loads (the highest among the cranial nerves and the second highest at all), which were comparable to the amount of viral copies in the amygdala (limbic system) and thalamus. Both structures are known to receive neuronal input via specific circuits from the olfactory system directly. The olfactory transmission route was shown to be very likely in horses suffering from Borna disease due to BoDV-1 ([34] and cited therein), and in horses a spread of BoDV-1 lesions is seen in the limbic system first [6]. The same transmission route was successfully used in rodent experiments before, showing intra-axonal transport of BoDV-1 [35]. Given the peak amounts of BoDV-1 in the olfactory bulb of the boy—especially when compared to the other cranial nerves—a centripetal and not a centrifugal viral spread along the nerve and thus an olfactory portal of entry seems probable. Whether this implies contact of BoDV-1-contaminated soil with the nose in the case described remains speculative. To further corroborate this finding, more cases should be analyzed for viral loads in the olfactory bulb in comparison to other cranial nerves and brain areas.

### Epidemiological considerations

A recent interview-based and case-controlled study showed that not a single specific transmission event could be recognized so far in 20 confirmed BoDV-1 cases [10], limited by the fact that relatives and not patients could be interviewed. The same holds true for the two pediatric cases here and thus, an indirect transmission via shrews´ secretions remains likely. Detailed epidemiological investigations of the cases did not suggest a common source of infection and sequencing confirmed two different virus strains, while the daily activities and the restricted range of motion of both children support the idea of peridomestic infection [10–12]. Without the knowledge of a specific transmission setting, a targeted prevention strategy is not possible with the exception of awareness campaigns for the disease and its natural host. However, outdoor activities near the patient’s homes had been assumed as risk factor in BoDV-1 endemic areas before [11], and an environmental source (such as soil contaminated with virus from shrew excreta) is likely in the absence of direct shrew contact as recently discussed [10]. A limited set of soil samples were therefore already analyzed for BoDV-1 RNA from patient’s gardens, but no virus could be detected [10]. In contrast, BoDV-1 was found in shrews previously in several endemic regions, and patient and shrew viral sequences often cluster together [11]. Comparing the children-derived BoDV-1 sequences with virus sequences from local animals (shrews or dead end hosts) could further support the epidemiological assumption of peridomestic transmission in these two cases as also discussed generally in previous epidemiological and phylogenetic studies [10–12]. The role of cats, predators of shrews, as possible transmission vectors is unclear but contact to cats could so far not be shown to be associated with BoDV-1 encephalitis [10]. Thus, sampling of natural hosts for BoDV-1 presence close to patients’ homes including studies on shrew population density, range and behavior, as well as shrew BoDV-1 infection status, seroprevalence studies among pets (cats) and farm animals, and extensive soil testing for viral RNA appear to be good strategies to better define risk settings. In contrast, serocontact and seroprevalence studies among humans including seroprevalence studies among children from the same area do not seem to be very useful: relatives in the two cases presented here, as well as household members in other cases [36] were seronegative, as were animal keepers at an alpaca farm with a recent BoDV-1 outbreak among the animals [19]. As the time of seroconversion appears variable with patients being seropositive at the time of hospitalization or shortly before death of encephalitis [4, 11, 12], serological testing during the incubation period is likely negative. This is exemplified here in Case 2 with an observed seroconversion on day 12 of clinical disease. In addition, a large seroprevalence study mainly in endemic regions with persons assumed to be at risk found only one seropositive individual (in whom a follow-up was not possible) among 1,109 participants [37]. Again, this reflects the rareness and sporadity of human BoDV-1 disease and, together with the finding of bornavirus-reactive antibodies based on the validated testing scheme only in encephalitic patients [12], a high manifestation index of clinical disease.

The only proven risk factor so far is living rurally, in small villages on the fringe of a settlement close to, or surrounded by, nature in virus-endemic regions [10]. Such places of residence showed a high odds ratio of 11 when matched and compared to rural, non-BoDV-1 cases in multivariable analysis [10]. Of note, the two cases described here lived in exactly such a surrounding, and even less than 1 km apart from each other, but on opposite sides of the village. Whether the geographic accumulation of human cases as currently experienced in other regions of Germany (unpublished data) simply reflects endemicity or whether this might be “hot spots “, needs to be further evaluated. Cases so far appeared with a time lag of several years, as also seen here. Interestingly, the two recovered BoDV-1 full genomes of the two pediatric cases here are not identical. They belong to the same viral cluster, but show striking divergence, thus reflecting different sources of infection (e.g., geographical micro-localities), temporal variation, or rather both. Epidemiological information about the two cases with different geographical exposures in their daily life supports this hypothesis. In any case, viral diversity in natural hosts is present in the area. We here defined the local cluster geographically, within the boundaries of a community, in this case a village with around 2000 inhabitants and an airline distance of the two cases less than 1 km, irrespective of viral diversity. The cluster led to the respective attention of the two cases by physicians, authorities, locals, and last but not least the media.

## Conclusion

Despite a very early diagnosis owing to increased awareness of BoDV-1 encephalitis among physicians and the general population due to the occurrence of a primary, also pediatric case in the same village, the outcome of the second case was fatal. The two cases described here illustrate that in face of immediate and intensive treatment attempts, no effective treatment regimen (unknown antiviral dosing and CSF penetration data; unclear use of additional immunomodulatory drugs) exists for this so far nearly uniformly fatal disease.

While living rurally in endemic areas, especially close to nature on the fringe of a settlement, is now a known risk factor [10], the exact transmission setting is unclear. The olfactory route is probably the portal of entry and future studies will have to address this in more detail. During the course of disease, viral RNA may be detected in excreted bodily fluids and barrier nursing is then recommended. Continuing prospective studies and surveillance will show whether case clusters reflect hot spots or endemicity. So far, prevention by education is of utmost importance as long as the gaps on knowledge prevail.
